# Carbapenem-Resistant *Salmonella* Typhi Infection in Traveler Returning to Germany from India, 2024

**DOI:** 10.3201/eid3112.251234

**Published:** 2025-12

**Authors:** Sandra Simon, Eva Trost, Jan Lennings, Julia Enkelmann, Julia Kuhn, Michael Pietsch, Antje Flieger

**Affiliations:** Robert Koch Institute, Wernigerode, Germany (S. Simon, E. Trost, M. Pietsch, A. Flieger); Public Health Department of Stuttgart, Stuttgart, Germany (J. Lennings); Robert Koch Institute, Berlin, Germany (J. Enkelmann); Ministry of Social Affairs, Health and Integration Baden-Wuerttemberg, Stuttgart (J. Kuhn)

**Keywords:** Salmonella, carbapenem-resistant, antimicrobial resistance, bacteria, bacterial infection, *Salmonella* Typhi, typhoid, typhoid fever, Germany, India

## Abstract

We report on a carbapenem-, extended spectrum β-lactam-, fluoroquinolone-, and tetracycline-resistant *Salmonella enterica* serovar Typhi strain in a patient returning to Germany from India. Considering the recent emergence of extensively drug-resistant *Salmonella* Typhi strains, further expansion of antibiotic resistance to carbapenems poses a serious threat for typhoid fever treatment.

Extensively drug-resistant (XDR) *Salmonella enterica* serovar Typhi, belonging to the H58 haplotype, was first identified in Sindh, Pakistan, in 2016 (*1*). Since then, those strains have been reported worldwide, mainly in association with travel to Pakistan. XDR *Salmonella* Typhi exhibit a multidrug-resistant (MDR) phenotype, including resistance to chloramphenicol, ampicillin, and sulfamethoxazole/trimethoprim, along with additional resistance to fluoroquinolones and third-generation cephalosporins. Consequently, therapeutic options for treating infections caused by XDR strains are primarily limited to the macrolide azithromycin and carbapenems. *Salmonella* Typhi strains resistant to carbapenems, azithromycin, or both have been reported occasionally. Carbapenem-resistant strains isolated in Pakistan harbored genes encoding VIM, GES, or NDM-5 carbapenemases (*2*,*3*). The respective NDM-5–positive strain showed the XDR phenotype and was phylogenetically assigned to the H58 haplotype. Recently, another case study described a non–XDR NDM-5–producing *Salmonella* Typhi isolate from India, which revealed additional resistance to fluoroquinolones and third-generation cephalosporins but remained susceptible to chloramphenicol, sulfamethoxazole/trimethoprim, and azithromycin (*4*).

We report an NDM-5–producing *Salmonella* Typhi strain isolated from a patient from Germany after returning from India. The patient, an experienced traveler to India who was last vaccinated with the typhoid polysaccharide vaccine in June 2021, undertook a 4-week round trip through several states in southwest India in September and October 2024. Upon return to Germany, the patient had onset of mild gastrointestinal symptoms, including diarrhea and abdominal pain. Symptoms gradually worsened over 2 weeks, prompting the patient to seek outpatient medical attention at a medical practice and a clinic, where stool and blood samples were collected. Blood tests were suggestive of a bacterial infection, and an empiric 3-day course of ciprofloxacin was commenced. However, after completion of the antibiotic therapy, the patient’s condition further deteriorated. Specifically, the patient had onset of fever (38.5°C) and severe headaches. Molecular stool diagnostics provided positive PCR signals for *Salmonella* and *Shigella* spp., but stool culture only resulted in growth of *Salmonella* spp. We subtyped the retrieved isolate (no. 24-09143) as *Salmonella* Typhi; phenotypic antimicrobial susceptibility testing according to European Committee on Antimicrobial Susceptibility Testing guidelines determined resistance to fluoroquinolones, tetracyclines, and β-lactam antibiotics, including penicillins, third-generation cephalosporins, and carbapenems (Table). The strain was susceptible to chloramphenicol, sulfamethoxazole/trimethoprim, and azithromycin, distinguishing it from MDR or XDR *Salmonella* Typhi and making appropriate treatment possible. The patient’s illness was successfully treated with a 14-day course of oral sulfamethoxazole/trimethoprim, and the patient made a full clinical recovery without requiring hospitalization. Three follow-up stool samples remained culture-negative.

**Table:** Antimicrobial-resistance profiles and corresponding genetic determinants of isolate 24-09143 from a traveler returning to Germany from India in comparison to selected published *Salmonella* Typhi genomes from Pakistan and India*

Antimicrobial class or substance	Genome and applied substance panel										
	24-09143 Germany 2024,† carbapenemase+, ESBL+			1790125 Pakistan 2022,‡ XDR+, carbapenemase+, ESBL+			Gurgaon01 India 2019,§ ESBL+			IOB-SWH-1 India 2024,# carbapenemase+	
	MIC, mg/L	Gene or mutation		MIC, mg/L	Gene or mutation		MIC, mg/L or DD, mm	Gene or mutation		MIC, mg/L or DD, mm	Gene or mutation
Penicillins											
Ampicillin	**>32**	*bla*_NDM-5_		**32**	*bla*_NDM-5_, *bla*_TEM-1_		**>256**	*bla*_TEM-1_		**>32**	*bla*_NDM-5_
Cephalosporins											
Cefotaxime	**>8**	*bla*_NDM-5_, *bla*_CTX-M-15_		**64**	*bla*_NDM-5_, *bla*_CTX-M-15_		NS	*bla*_CTX-M-15_		NS	*bla*_NDM-5_
Ceftazidime	**>8**			**128**			**>256**			NS	
Cefoxitin†	**>32**			**64**			NS			NS	
Ceftriaxon	NS			NS			**>256**			**>64**	
Quinolones											
Ciprofloxacin	**2**	*gyrA_*S83Y, *qnrS1*		**64**	*gyrA_*S83F, *qnrS1*		**2**	*gyrA_*S83Y, *qnrS1*		**>4**	*gyrA_*S83Y
Nalidixic acid¶	>32			8			DD 6 mm			NS	
Carbapenems											
Meropenem	**>16**	*bla*_NDM-5_		**16**	*bla*_NDM-5_		0.023	NS		**>16**	*bla*_NDM-5_
Macrolides											
Azithromycin¶	2	NS		**64**	*mphA*		8	NS		6	NS
Phenicols											
Chloramphenicol¶	4	NS		**64**	*catA*		DD 28 mm	NS		DD (susceptible)	NS
Tetracyclines¶	**>32**	*tet*(A)		**32**	*tet*(A)		**64**	*tet*(A)		NS	NS
Sulfonamides¶	NS	NS		**512**	*sul-1*		NS	*sul-2*		NS	NS
Trimethoprim	0.5	NS		**16**	*dfrA-27*, *dfrA-7*		NS	dfrA14		NS	NS
Sulfamethoxazole/trimethoprim	<0.25	NS		NS	*sul-1*, *dfrA-27*, *dfrA-7*		**>256**	*sul-2*, *dfrA14*		<20	NS

*Bold indicates resistance. DD, disk diffusion assay; ENA, European Nucleotide Archive; ESBL, extended-spectrum β-lactamase; NS, not specified; XDR, extensively drug-resistant; +, positive. †ENA accession no. ERR15390137. ‡ENA accession no. SRR22801766. §ENA accession no. ERR3527963. #ENA accession no. SRR32461882. ¶No clinical breakpoints defined by European Committee on Antimicrobial Susceptibility Testing.

We performed whole-genome analysis by using Illumina (https://www.illumina.com) (European Nucleotide Archive [ENA] accession no. ERR15390137) and Oxford Nanopore (https://nanoporetech.com) ( accession no. ERR15647836) technologies. We compared resistance determinants of strain 24-09143 from the reported case with published *Salmonella* Typhi strains from Pakistan showing XDR, azithromycin, and carbapenem resistance (*3*), and from India, characterized by either extended-spectrum β-lactamase (ESBL) (*5*) or NDM-5 production (*4*) (Table). Resistance determinants of strain 24-09143 included *tet*(A) for tetracycline, *qnrS1* for quinolone, *bla*_CTX-M-15_ for cephalosporin, and *bla*_NDM-5_ for carbapenem. On the basis of the hybrid assembly of the long and short read data (obtained by using unicycler version 0.4.8, https://github.com/rrwick/Unicycler), we located the resistance genes on 2 putative conjugative plasmids of replicon types IncFIB (*qnrS1* and *tet*(A), *bla*_CTX-M-15_, 73.3 kb) and IncX3 (*bla*_NDM-5_, 46.2 kb). *bla*_CTX-M-15_ is regularly found in ESBL-producing *Salmonella* Typhi of the Pakistan XDR sublineage, either plasmidborne or integrated in the genome (*1*,*6*), and has also been described in non–XDR *Salmonella* Typhi from India (*5*,*7*). Although carbapenemase production has thus far been observed rarely in *Salmonella* Typhi (*3*,*4*), IncX3 plasmids carrying *bla*_NDM-5_ have been reported in nontyphoidal *Salmonella* (*8*,*9*) and in other enterobacterial genera.

Phylogenetic analysis assigned the strain to the H58 haplotype but distinguished it from the previously described NDM-5–positive XDR *Salmonella* Typhi from Pakistan (strain no. 1790125; ENA accession no. SRR22801766) (*3*) by 22 single-nucleotide polymorphisms (SNPs) (Figure). However, the analysis revealed a very close relationship to the recently described NDM-5–positive isolate from India (0 SNPs; strain no. IOB-SWH-1; ENA accession no. SRR32461882) (*4*) and to a representative of a carbapenemase-negative ESBL-producing *S*. Typhi lineage also orginating from India (*5*) (4 SNPs; strain Gurgaon01; ENA accession no. ERR3527963) (Figure). The presence of the *bla*_NDM-5_ gene in representatives of different *Salmonella* Typhi sublineages presumably indicates independent acquisitions in the XDR sublineage from Pakistan and an ESBL-producing sublineage from India, which might increase the risk for global dissemination of carbapenem resistance in which transmission is enabled by travel and migration. This risk is exemplified for the reported case, in which an individual contracted the NDM-5–producing *Salmonella* Typhi while traveling in India and brought the strain to Germany upon return. Alarmingly, the increasing occurrence of XDR, ESBL-producing, and carbapenemase-producing strains severely limits effective treatment options for typhoid fever. This finding emphasizes the need for comprehensive antimicrobial susceptibility testing of clinical *Salmonella* Typhi isolates to ensure appropriate treatment, while avoiding the use of last-line antibiotics when they are not necessary. It also highlights the importance of prevention measures, such as improved sanitation, access to clean water, and typhoid vaccination (or revaccination).

**Figure:**
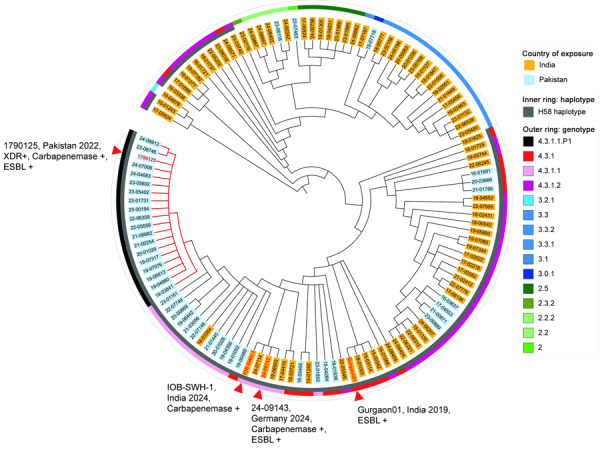
Phylogenetic structure of *Salmonella enterica* serovar Typhi strain 24-09143 from a traveler returning to Germany from India and maximum-likelihood tree based on 351 single-nucleotide polymorphisms and visualized with Interactive Tree Of Life ([https://itol.embl.de) showing phylogenetic structure of *Salmonella* Typhi isolates from Pakistan and India. Tree includes 168 isolates from the *Salmonella* Typhi surveillance program of Germany’s Reference Center for *Salmonella* and other Bacterial Enteric Pathogens with reported case exposure in India or Pakistan (2015–2024) and three selected published genomes of strains from these countries. Strain 24-09143 belongs to the H58 haplotype and is closely related to previously described *bla*_NDM-5_– or *bla*_CTX-M-15_–positive *Salmonella* Typhi strains from India, specifically strain IOB-SWH-1 from 2024 and strain Gurgaon01 from 2019. It is clearly distinguishable from the XDR H58 sublineage from Pakistan, such as *bla*_NDM-5_– and *bla*_CTX-M-15_–positive strain 1790125 from 2022. Isolate labels are colored according to the country of origin or exposure. Red type indicates branches corresponding to the XDR sublineage from Pakistan (genotype 4.3.1.1 P1). Inner ring shows the H58 haplotype in gray, and outer ring depicts the genotypes described by Wong et al. (*10*). Red arrowheads indicate carbapenemase- and ESBL- producing isolates referred to in the study and Table. ESBL, extended-spectrum β-lactamase; XDR, extensively drug-resistant.
